# Will E-Cigarette Modified Risk Messages with a Nicotine Warning Polarize Smokers’ Beliefs about the Efficacy of Switching Completely to E-Cigarettes in Reducing Smoking-Related Risks?

**DOI:** 10.3390/ijerph18116094

**Published:** 2021-06-05

**Authors:** Bo Yang, Juliana L. Barbati, Yunjin Choi

**Affiliations:** Department of Communication, University of Arizona, Tucson, AZ 85721, USA; jbarbati@email.arizona.edu (J.L.B.); ychoi@email.arizona.edu (Y.C.)

**Keywords:** modified risk claims, e-cigarettes, nicotine addictiveness warning, opinion polarization, need for closure

## Abstract

In the U.S., e-cigarette companies can apply for permission to use reduced or modified risk messages (MRMs) in their marketing materials. Because e-cigarette marketing materials should have a nicotine addictiveness warning, MRMs and a nicotine warning could appear together—resulting in a conflicting message. When reading a conflicting message, individuals assimilate evidence supporting their pre-existing beliefs and eventually develop stronger beliefs, diverging more from those with different pre-existing beliefs (i.e., polarization). This study examined if exposure to e-cigarette MRMs with a nicotine warning polarizes smokers’ initially opposing beliefs about the efficacy of switching completely to e-cigarettes in reducing smoking-related risks, and if this polarization depends on individuals’ need for closure. An online experiment randomized 761 U.S. adult smokers to either three MRMs with a nicotine warning or three control messages. People reported their perceived efficacy of switching completely to e-cigarettes at pre- and posttest and need for closure at pretest. Linear regression showed no polarization effects. Nonetheless, need for closure and pretest efficacy beliefs influenced message response: MRMs with a nicotine warning only enhanced efficacy beliefs of smokers with low pretest efficacy beliefs and low need for closure. Evaluation of e-cigarette mixed communication should consider individuals’ motivational and cognitive differences.

## 1. Introduction

### 1.1. Background

After being introduced to the U.S. market in 2007, e-cigarettes quickly gained popularity. While the long-term health effects of e-cigarettes await more research, existing evidence suggests that e-cigarettes may exude fewer toxins compared to combustible cigarettes [1,2,3,4,5]. As a result, there has been more discussion in recent years about encouraging a complete switch to e-cigarettes among smokers who otherwise are unable or unwilling to quit smoking [6,7,8,9]. Supposedly, this could reduce smokers’ smoking-related health risks.

In the U.S., tobacco companies can submit applications (i.e., Modified Risk Tobacco Product Applications or MRTPAs) to the U.S. Food and Drug Administration (FDA) for using reduced or modified risk claims in their marketing materials [10]. The FDA thus far has received MRTPAs for several smokeless tobacco products (General Snus, Copenhagen, and Camel Snus), an electronic heated tobacco product (IQOS), and a reduced nicotine cigarette brand (VLN™ King) [11]. By March 2021, the FDA has permitted General Snus to use modified risk claims stating that their products pose lower levels of smoking-related risks than cigarettes and IQOS to claim that switching to their products reduces smokers’ exposure to harmful substances [12]. E-cigarettes have been a central subject of the tobacco harm reduction discussion [6]. Following other tobacco products, e-cigarette MRTPAs may happen in the future. Thus, it is important to understand how consumers react to modified risk messages (MRMs) about e-cigarettes.

In the current study, we focus on the effects of e-cigarette MRMs combined with a nicotine addictiveness warning label. In August 2018 [13], the FDA required that e-cigarette promotion materials include a warning label indicating that e-cigarettes contain nicotine, an addictive chemical. Thus, an MRM might appear in tandem with a nicotine warning. However, a combination of an MRM with a nicotine warning could result in a conflicting message (i.e., a message that both supports and opposes the same subject) and could be perceived as ambiguous [14]. Both qualitative [15,16,17] and quantitative studies [18] have documented such a possibility.

Conflicting health messages may adversely influence people’s knowledge and beliefs [14,19,20,21,22] about a health issue. For instance, Nagler and colleagues [20] found that exposure to conflicting recommendations about mammography increased people’s confusion, negative affective responses, and negative reactions towards research and recommendations about cancer prevention. Similarly, Chang [22] found that news presenting conflicting evidence about health effects of drinking milk and jogging produced decreased message credibility, less positive attitudes towards health research, greater uncertainty, and weaker message compliance intentions.

A few studies have also examined e-cigarette conflicting communication. A national survey [23] in 2014 found that U.S. adults’ exposure to conflicting information about e-cigarettes was associated with weaker support for several e-cigarette regulation policies, including a policy about enforcing an addiction warning label. An experimental study [18] among U.S. nonsmokers found that the addition of MRMs to an e-cigarette warning on e-cigarette packaging increased perceived message ambiguity, and in turn reduced perceived warning effectiveness, as well as intentions to stay away from e-cigarettes. In another experiment [24], 2056 U.S. adults reported no differences between conflicting e-cigarette messages and control messages whereas positive and negative e-cigarette messages produced responses consistent with the messages. Very recently, scholars [25] found that personal characteristics impacted people’s responses to e-cigarette messages mentioning both positive and negative product effects. Specifically, among those with a low information avoidance tendency, exposure to conflicting e-cigarette information produced more confusion than one-sided anti-e-cigarette messages or control messages. Among those with a high information avoidance tendency, however, message effects did not differ.

Overall, past studies have delivered some useful insights into how people respond to e-cigarette messages when they include both supportive and opposing information. However, no studies have considered the role of individuals’ initial beliefs about a message topic (i.e., e-cigarettes in the present context)—a very important factor shaping mixed or conflicting message responses according to the communication and psychology literature [26,27]. Our study aims to perform a preliminary examination of this issue. Specifically, based on the biased assimilation and opinion polarization literature, we examined if smokers who initially differed in their perceived efficacy of switching completely to e-cigarettes in reducing smoking-related risks would become stronger in their beliefs after exposure to MRMs with a nicotine warning. Additionally, following Yang and colleagues [25] and prior studies on biased assimilation of mixed health communication [28,29], we examined another personality variable, need for closure, in the interplay of initial e-cigarette efficacy beliefs and MRMs with a nicotine warning on smokers’ posttest perceived e-cigarette efficacy in reducing smoking-related health risks.

### 1.2. Biased Assimilation and Opinion Polarization

When people are exposed to mixed or conflicting information, they tend to selectively attend to information that confirms their pre-existing beliefs, while ignoring or discounting information that challenges them [30,31]. For instance, upon exposure to messages including evidence that both supports and refutes the death penalty, supporters of the death penalty only attend to evidence in support of the death penalty while opponents only focus on the opposing evidence [30]. This phenomenon is termed biased assimilation. Biased assimilation posits that people’s initial stance on an issue will become stronger after being exposed to a conflicting message. This attitude strengthening leads to the gap between those initially disagreeing with each other to become wider—referred to as opinion polarization [30]. In other words, exposure to conflicting information will lead people to disregard the information that does not coincide with their pre-existing attitudes. Simultaneously, they will attend to the information that they already agree with, leading them to feel even stronger in their opinion. Eventually, people’s original opposing opinions diverge into two opposite extremes.

Biased assimilation and attitude polarization have been documented in various domains [30,32,33,34,35], including the health domain [28,29,36,37]. For instance, Kahan and colleagues [28] found that individuals who initially held opposing views towards the HPV vaccination mandate disagreed more with each other following exposure to messages including both pro- and counter-vaccine arguments (vs. a control message). Similar to Kahan and colleagues and more relevant to the present study, Ofori-Parku [36] presented a message mentioning both risks and benefits of smoking to college students who held cultural worldviews that either supported or opposed smoking. Following message exposure, researchers asked students to report perceived smoking risks. Results revealed that those who saw the mixed message (vs. those who did not) were more convinced of their original position after message exposure.

To the best of our knowledge, no study has tested biased assimilation and attitude polarization in the context of e-cigarettes. However, similar to previously examined issues (e.g., HPV vaccine) in the polarization literature, there are different beliefs and attitudes regarding e-cigarettes [38], particularly regarding their potential as a less harmful smoking alternative. As such, exposure to mixed e-cigarette messages may produce biased assimilation and divergent opinions. Specifically, according to prior empirical findings about biased assimilation and opinion polarization [28,29,36,39], smokers exposed to MRMs with a nicotine warning might choose to focus on different information depending on their initial efficacy beliefs about switching completely to e-cigarettes in reducing smoking-related risks. As a result, they may report polarized efficacy beliefs after message exposure. We make the following prediction:

**Hypothesis** **1** **(H1).** *Exposure to MRMs with a nicotine warning polarizes smokers who initially hold different beliefs about the efficacy of switching completely to e-cigarettes in reducing smoking-related risks such that: (a) exposure to MRMs with a nicotine warning (vs. control messages)* *increases efficacy beliefs among smokers with high efficacy beliefs at pretest; (b) exposure to MRMs with a nicotine warning (vs. control messages)* *decreases efficacy beliefs among smokers with low efficacy beliefs at pretest.*

We focused on people’s beliefs about the efficacy of switching to e-cigarettes in reducing smoking-related risks because tobacco companies’ modified risk marketing is very likely to focus on changing such a belief. For example, the MRM from IQOS says: “Switching completely from conventional cigarettes to the IQOS system significantly reduces your body’s exposure to harmful or potentially harmful chemicals” [40]. Additionally, beliefs about the efficacy of an issue in addressing a problem have been an important outcome in prior attitude polarization literature, such as the original study on this concept (i.e., the perceived deterrent efficacy of the death penalty) [30].

### 1.3. Need for Closure

One important goal of modified risk communication is to convey knowledge about a modified risk tobacco product so that consumers can make more informed decisions [10]. However, individuals differ from each other in their knowledge acquisition motives [41]. Such differences may influence the processing of modified risk information. As a result, evaluation of modified risk communication should consider individuals’ differences in their knowledge acquisition motives. In this study, we examined need for closure—a major motivational factor governing knowledge acquisition and an important individual difference variable that impacts attitudes and attitude change [42].

Need for closure describes “individuals’ desire for a firm answer to a question and an aversion toward ambiguity” [43] (p. 264). The construct comes from Kruglanski’s lay epistemic theory [41]. Lay epistemic theory examines the process through which people acquire and modify their knowledge; this theory has received much discussion in the persuasion and attitude change literature [44].

Individuals’ need for closure can be influenced by situational factors, such as time constraints, rewards for being closed-minded, environmental noise, and mental fatigue (i.e., fatigue after prolonged cognitive activity). Additionally, the construct is known to vary as an individual difference variable [45]. Individuals high on need for closure prefer order, predictability, and decisiveness, but dislike ambiguity and inconsistent information. As a result, high need-for-closure people usually seek prompt and permanent cognitive closure. While processing information and making decisions, those high on need for closure “seize” quickly on accessible cues and then “freeze” existing beliefs and resist new information [43]. Compared with those high on need for closure, individuals low on need for closure have increased open-mindedness. They consider various perspectives before making a judgment [43]. These differing tendencies among high and low need-for-closure people result in a wide range of social-cognitive phenomena [46]. For instance, high need-for-closure people report greater use of readily accessible information, preference for prototypic (vs. diagnostic) information, and greater confidence in subjective judgments.

The differences between people with low and high need for closure in information processing and tolerance of ambiguity suggest that need for closure could influence how individuals respond to conflicting messages. Indeed, Nan and her colleague [29] asked college students to view blogs containing positive and negative opinions about the HPV vaccine and assessed students’ chronic need for closure. They found that need for closure worked in tandem with students’ beliefs about the effectiveness of vaccinations in general to influence students’ responses to the conflicting blogs. Specifically, among only individuals with high need for closure, opinion polarization occurred: those with high initial vaccination effectiveness perceptions reported higher HPV vaccine effectiveness, while those with low initial perceptions reported lower HPV vaccine effectiveness after being exposed to the conflicting blogs. Nan and her colleague’s study revealed that the addition of need for closure can lead to a better understanding of belief polarization.

Extending upon Nan and her colleague’s study, we chose to explore whether need for closure moderates the polarization effects predicted in H1. Additionally, need for closure is an important epistemic motivational variable [42] and may play an important role in the perceptions of modified risk communication and advertisements. Recently, scholars [25] found that smokers’ information avoidance tendency was closely related to their responses to e-cigarette mixed messages. Need for closure has long been discussed as an important variable related to individuals’ information acquisition tendencies and behaviors [45,47,48,49]. By examining need for closure, we logically extended previous findings regarding the role of individual difference variables in shaping responses to mixed e-cigarette messages. Informed by Nan and her colleague’s study [29], we predict:

**Hypothesis** **2** **(H2).** *The effects described in H1 will be more pronounced among smokers with high (vs. low) need for closure.*

## 2. Materials and Methods

### 2.1. Design, Participants, and Procedure

This study is part of a larger experiment on e-cigarette and nicotine risk communication. Specifically, the whole experiment included five message conditions (*n* = 1906; for a detailed description of the five conditions, see our earlier publications [50,51]). In the current study, we analyzed data from only two of the five message conditions relevant to our hypotheses (*n* = 761; discussed below). The other three conditions in the larger experiment were excluded.

The sample (*n* = 761; ≥18 years old) consisted of primarily current smokers who reported having consumed at least 100 cigarettes in their life, and currently used cigarettes “every day” or “some days”. The sample also had a small number of former smokers who had consumed at least 100 cigarettes in their life and had quit smoking in the past two years. Similar to previous tobacco communication and behavior research [52,53,54,55], we recruited participants from Toluna, a commercial research company. Toluna has “the worlds’ largest online social voting community” (p. 1) [56], with more than 36 million registered participants available for various types of online surveys around the world. In the U.S., Toluna reserves nearly 7 million people who are recruited through various strategies (e.g., web banners, website referrals, pay-per-click, affiliate marketing, and email). Toluna aims to create a diverse participant community similar to the general population distribution (for more data quality information, see https://tolunacorporate.com/wp-content/uploads/2020/07/ID_080-Quality-eBook_v02-1.pdf) (accessed on 3 June 2021). Upon panel registration, Toluna collects participants’ personal information, including tobacco use history. Once a study becomes available, Toluna randomly selects eligible participants to contact about the study. For our study, participants needed to meet the age and smoking behavior requirements specified above. Participants who completed our study received points that could be redeemed for cash or vouchers for store credit.

The study was implemented on Toluna’s web portal. Participants first completed an electronic consent form. In the experiment, participants answered questions about their demographics, their tobacco use, and beliefs about e-cigarettes, including their pre-existing perceived efficacy of switching completely to e-cigarettes in reducing smoking-related health risks. Then, they were randomly assigned to a message condition. To reiterate, the purpose of the present study is to explore whether MRMs with a nicotine warning have polarization effects. In addressing similar questions, previous biased assimilation and polarization research has compared a mixed message condition with a control condition (e.g., no message or a brief message without arguments for each side) [28,29,36,39]. Additionally, the FDA requires e-cigarette marketing materials to include a nicotine warning label. As such, when smokers see MRMs in e-cigarette modified risk marketing or advertising, most likely they will see the messages in tandem with a nicotine warning label instead of MRMs alone. Based on this logic and previous research, we selected only two conditions from the larger experiment: (1) MRMs with a nicotine warning condition and (2) a control condition. In each condition, participants read three messages one after another in a random order. After all messages were shown, participants reported outcome measures, including their perceived efficacy of switching completely to e-cigarettes in reducing smoking-related risks.

The study was conducted in accordance with the Declaration of Helsinki, and its protocol was approved by Georgia State University’s Institutional Review Board (IRB number: H18570). All participants received a debriefing message and were directed to smoking cessation resources at the end of the study.

### 2.2. Message Stimuli

The MRMs with a warning condition included three MRMs plus the same FDA requested nicotine warning label. The MRMs across three messages aimed to communicate that switching completely to e-cigarettes could reduce smokers’ risks for smoking-related diseases, and if smokers cannot quit smoking, they can instead switch completely to e-cigarettes. We developed the message content through a review of existing e-cigarette messages and e-cigarette health risk literature as well as focus group discussions (for detailed message development, please see our other publications [16,57]). The MRMs were delivered in a format informed by existing MRMs submitted by tobacco companies, as well as our own knowledge about e-cigarette advertisements. The nicotine warning label was designed and formatted according to the FDA’s standards. It reads: “This product contains nicotine. Nicotine is an addictive chemical.” The control condition included three bottled water ads. Similar to the modified risk stimuli, bottled water ads each had textual information placed against a large visual background (for detailed stimuli, see the Supplementary Material Figure S1). The amount of time spent viewing the message stimuli did not differ based on condition, *F* (1, 759) = 0.65, *p* = 0.42, η^2^ = 0.001.

### 2.3. Key Measures

Pretest and posttest perceived efficacy of switching completely to e-cigarettes were assessed with the same scale [58]. Before and after message exposure, participants reported their level of agreement with three items on a 1 to 9 scale: “Switching completely to e-cigarettes is effective at reducing my chances of getting cancer”, “If I switch completely to e-cigarettes, I am less likely to get a serious disease”, and “If I switch completely to e-cigarettes, I will have fewer health risks”. We averaged ratings across these three items to create a composite score for pretest (perfect model fit due to a saturated model; M = 4.73, SD = 0.95, α = 0.94) and posttest perceived efficacy (saturated model; M = 5.19, SD = 2.44, α = 0.96).

Need for closure was assessed with a pre-established scale [59]. Participants indicated their level of agreement across 15 items on a 1 to 10 scale. Example items included “I don’t like situations that are uncertain”, “I dislike questions which could be answered in many different ways”, and “I dislike unpredictable situations”. Ratings of the 15 items were averaged to create a composite score (one-factor model: χ^2^(75) = 413.99, *p* < 0.001, RMSEA = 0.077, 90% CI [0.070, 0.084], SRMR = 0.056, CFI = 0.903; M = 5.03, SD = 2.48, α = 0.87).

Control variables assessed and controlled for included gender, race/ethnicity, age, education, nicotine dependence [60,61], past quitting attempt, current and ever e-cigarette use, and ever switch to a lower tar or nicotine cigarette.

### 2.4. Analysis Plan

To test our hypotheses, we performed one ordinary least square regression analysis. The regression model had posttest perceived efficacy of switching completely to e-cigarettes as the dependent variable and controlled for the covariates listed above. Key independent variables included message condition (MRMs vs. control), pretest perceived efficacy of switching completely to e-cigarettes, and need for closure, as well as their two- and three-way interactions. To avoid multicollinearity, composite scales in the interaction terms were mean-centered before forming products.

Our H1 predicted that (a) exposure to MRMs with a nicotine warning increases efficacy beliefs among smokers with high pretest efficacy beliefs and (b) exposure to MRMs with a nicotine warning decreases efficacy beliefs among smokers with low pretest efficacy beliefs. This implies a significant two-way interaction between pretest perceived efficacy of switching completely to e-cigarettes and message condition. H2 predicted that the prediction posed in H1 should be more pronounced amongst smokers with high (vs. low) need for closure, implying a three-way interaction among pretest perceived efficacy, message condition, and need for closure. Significant interaction terms were probed with Hayes’ PROCESS macro [62] to identify whether the patterns were consistent with H1 and H2. Following Hayes’ suggestions, to test H1, we explored the relationship between message condition and posttest perceived efficacy at two values (high: M + 1SD; low: M − 1SD) of pretest perceived efficacy. To test H2, we explored the two-way interaction between message condition and pretest perceived efficacy at high (M + 1SD) and low levels (M − 1SD) of need for closure. Data analyses were conducted in Stata v14.1 and SPSS v24.

## 3. Results

### 3.1. Sample Characteristics

Table 1 lists the sample characteristics. According to Table 1, amongst all participants, 51.5% identified as female, 73.9% identified as White, and 50.3% had at least some college education. The majority of participants smoked daily (63.3%), and more than half had attempted to quit smoking in the past year (52.6%). Nearly 50% were currently using e-cigarettes. The sample included similar numbers of people from different age groups (M = 43.77, SD = 15.51).

### 3.2. Hypothesis Testing

**Figure 1:**
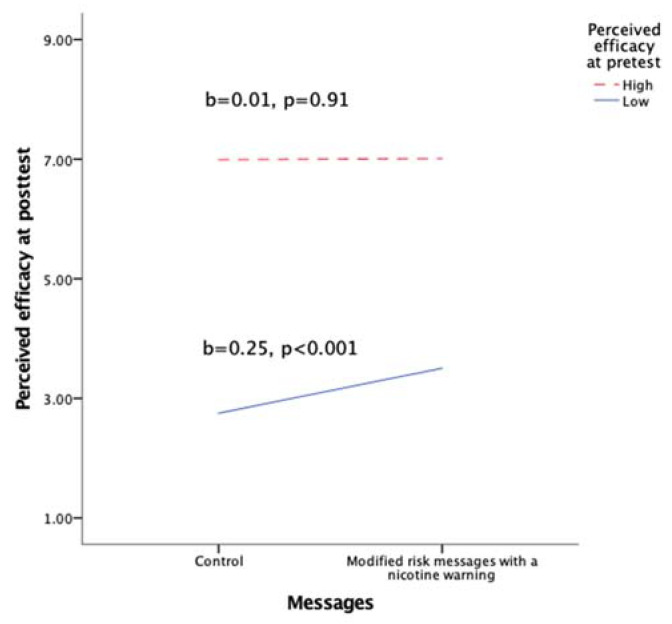
Two-way interaction between message condition and pretest perceived efficacy of switching completely to e-cigarettes in reducing smoking-related risks.

Results are shown in Table 2 and Figure 1. MRMs produced higher perceived efficacy of switching completely to e-cigarettes than the control messages (b = 0.12, *p* = 0.002, η^2^ = 0.005, *r* = 0.07). However, this main effect was further qualified by a two-way interaction (b = −0.04, *p* = 0.007, η^2^ = 0.004, *r* = 0.06). Specifically, as shown in Figure 1, amongst individuals who held high levels of efficacy beliefs at pretest, peoples’ posttest perceived efficacy did not differ based on message condition (b = 0.01, *p* = 0.91); amongst individuals who held low levels of efficacy beliefs at pretest, MRMs produced higher posttest perceived efficacy than the control messages (b = 0.25, *p* < 0.001). Because this pattern was inconsistent with H1, H1 was rejected.

**Figure 2:**
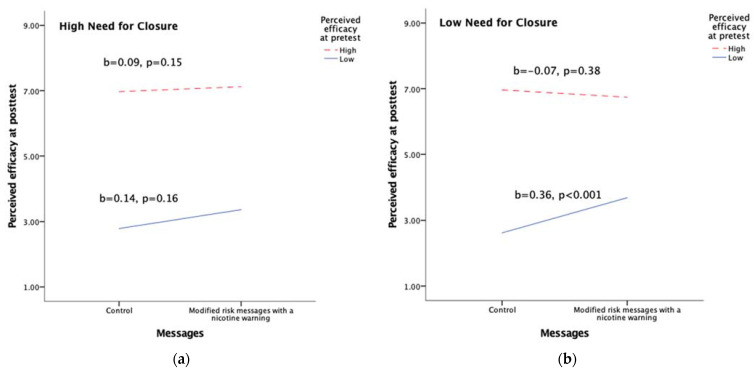
Three-way interaction between message condition, need for closure, and pretest perceived efficacy of switching completely to e-cigarettes in reducing smoking-related risks: (**a**) high need for closure; (**b**) low need for closure.

In testing H2, we found a significant three-way interaction (b = 0.04, *p* = 0.01, η^2^ = 0.003, *r* = 0.06; Table 2). Further exploration (see Figure 2) revealed that amongst individuals with high levels of need for closure, regardless of their levels of efficacy beliefs at pretest, MRMs did not yield different efficacy beliefs compared to the control messages (high: b = 0.09, *p* = 0.15; low: b = 0.14, *p* = 0.16). Amongst individuals with low levels of need for closure, when they held high levels of pretest efficacy beliefs, exposure to MRMs did not influence their efficacy beliefs (b = −0.07, *p* = 0.38); when individuals held low levels of pretest efficacy beliefs, exposure to MRMs increased their perceived efficacy of switching completely to e-cigarettes in reducing smoking-related risks compared to the control messages (b = 0.36, *p* < 0.001). This interaction suggests that message condition interacted with pretest efficacy beliefs only amongst those with low need for closure, and the interaction did not show belief polarization. H2 was rejected.

## 4. Discussion

We posed two hypotheses to test whether e-cigarette MRMs with a nicotine addictiveness warning label would polarize smokers’ beliefs about the harm reduction efficacy of switching completely to e-cigarettes (i.e., increase the divergence of pre-existing efficacy beliefs). We first predicted that after observing MRMs with a warning label (vs. control messages), smokers with differing pre-existing efficacy beliefs would increase in the strength of their pre-existing beliefs, resulting in a wider belief gap. Our findings did not support this prediction. Instead, we found that MRMs with a warning increased the efficacy beliefs of smokers with low pretest efficacy beliefs and had no effect on smokers with high pretest efficacy beliefs. Thus, MRMs with a warning actually narrowed the gap between smokers with different pre-existing efficacy beliefs.

Our second hypothesis introduced need for closure, an individual motivational variable that can influence the processing of conflicting information. We expected that smokers with high (vs. low) need for closure would be more likely to report belief polarization. We failed to support this hypothesis: amongst smokers with high need for closure, regardless of their pretest perceived efficacy about switching completely to e-cigarettes, MRMs with a warning did not influence their posttest efficacy beliefs. Amongst smokers with low need for closure, MRMs with a warning increased the efficacy beliefs of only those holding low pre-existing efficacy beliefs. This finding is similar to what we identified for H1, which suggests that it could be smokers with low need for closure and low pre-existing efficacy beliefs that drove our findings for H1.

Taking all the findings together, we are unable to conclude that exposure to MRMs with a nicotine warning polarizes smokers’ beliefs about the efficacy of switching completely to e-cigarettes in reducing smoking-related risks even when considering need for closure. Our findings are inconsistent with previous biased assimilation and belief polarization literature [28,29,37], which might be due to the following reasons.

First, smokers with high pretest efficacy beliefs might have experienced a ceiling effect. Thus, MRMs with a warning would not exert a significant effect on them. Second, people with high need for closure tend to have a greater risk aversion tendency [63]. Because e-cigarettes are widely known as a risky product [64], those high on need for closure were not influenced by the MRMs possibly due to a salient risk aversion tendency. Third, in past biased assimilation and polarization studies [28,29,30,34,35], the conflicting messages were usually more balanced in how they presented conflicting information. For example, in Nan and Daily’s study [29], the supportive and oppositional HPV blogs were equal in length and design. In the current study, our MRMs were much longer, more colorful, and conveyed more health effect evidence than the nicotine warning label. Additionally, the background visuals tended to be more consistent with the overall tone of the MRMs. This might have reduced people’s processing of the nicotine warning label and decreased the impact of the overall messages, affecting our capacity to produce findings consistent with former research. However, according to tobacco industry’s common advertising strategies and existing MRMs, real-world modified risk ads might be similar to our stimuli that will use visuals appealing to people’s positive emotions in support of MRMs. Future research may want to explore how message design factors influence the processing of MRMs and a health warning and the role of individuals’ cognitive and motivational factors in this process. Finally, our results may be a product of our sample, which only included smokers. It is possible that smokers perceived MRMs with a warning differently than nonsmokers. Indeed, Katz and colleagues [18] found that perceptions of ambiguity differed based on smoking status. Nonsmokers perceived MRMs with a warning as ambiguous, while smokers did not. The same might have occurred in our smoker sample. Additionally, smokers might have been engaged in motivated reasoning [65] and thus were more likely to focus on the portion of the messages that suggested they could continue using nicotine products. Due to the potential lack of perceptions of ambiguity in our sample, and the potential for motivated reasoning, our expected polarization effects were limited. Given the novelty of our hypotheses, to better explain our findings, we encourage future research to further examine these relationships.

While our results did not support the notion of MRMs with a nicotine warning leading to attitude polarization, amongst smokers with low pretest efficacy beliefs and low need for closure, MRMs with a nicotine warning increased posttest efficacy beliefs; nonetheless, for smokers high in need for closure, MRMs with a nicotine warning did not have any effect. This indicates that smokers with high need for closure and low pretest efficacy beliefs might have more heavily considered the information against e-cigarettes and were less swayed in the direction of the MRMs compared to their low need-for-closure counterparts.

Our study provides important practical implications. When the FDA reviews Modified Risk Tobacco Product Applications (MRTPAs) from tobacco companies, it is important to consider whether consumers can correctly understand tobacco companies’ promotional materials regarding modified risk [10]. In MRTPAs, modified risk claims usually appear with some warning. In this case, our study suggests that consumers’ understanding of modified risk from MRMs can be influenced by people’s cognitive and motivational attributes: specifically, their pre-existing beliefs about the modified risk statement and their need for closure. Our study found that MRMs with a warning had a main effect in enhancing beliefs consistent with the MRMs. However, when considering pretest efficacy beliefs and need for closure, we found that the main message effect was significant only amongst those with low pretest efficacy beliefs and low need for closure. The specific finding that MRMs with a warning had no influence amongst smokers with low pretest efficacy beliefs and high need for closure may indicate a missed harm reduction opportunity. Based on our findings, evaluation of MRMs with a warning should also consider consumers’ motivational and cognitive traits that might influence their understanding of the modified risk statements. Notably, while need for closure can be considered a stable trait, it can be impacted by certain situational variables [46]. For instance, situations including less time for decision making, high mental fatigue (i.e., fatigue after prolonged cognitive activity), perceived low attractiveness of a task, increased difficulty of information processing and comprehension, as well as noisy environments can induce high need for closure [46]. This suggests that how and where people process MRMs may influence people’s understanding of MRMs as it concerns need for closure. The FDA requires that tobacco companies provide sample marketing materials and describe their promotion strategies in their MRTPAs [10]. We suggest that such discussion may consider the specific marketing format and channel of MRMs that can influence consumers’ level of need for closure. For instance, direct mail and brochures might be designed to present a lot of information. By contrast, product packaging might show less information. Hence, processing direct mail and brochures vs. product packaging might be more cognitively demanding and can easily lead to cognitive closure. As a result, interpretations of MRMs and warning messages on direct mail and brochures may be different from those of product packaging. Specifically, high levels of need for closure induced by cognitively demanding materials might interfere with people’s understanding of modified risk information. As such, the FDA might also want to regulate the amount of information and design of modified risk marketing materials to make sure MRMs and warning information can be accurately understood.

Our study is limited in using a convenience sample. Although the sample is diverse in terms of demographics, it cannot accurately represent all U.S. smokers. Additionally, participants only viewed the stimuli for a short time in an artificial environment and reported their efficacy beliefs immediately following message exposure. Due to such a design, we cannot rule out any demand effects or assure that our findings remain in the long term. Future research can test messages using a longitudinal design that implements message exposure and measurement at different times [66]. Furthermore, due to the study design, we could not assess how our messages affect smokers’ behavior. Given behavioral change is an important focus of tobacco control interventions, future research should also include behavior as an outcome and assess how polarized beliefs influence tobacco use behaviors. In this study, we presented a set of three MRMs with different designs to smokers. Although this reduced the threat of presenting only a single message and mimicked real-life situations where smokers may see multiple messages about e-cigarettes, the fixed message content and dosage might still affect the validity of our findings [67]. Particularly, our manipulation of the MRMs included many visuals in a positive tone. Though they were designed based on our knowledge of e-cigarette advertising, such a design might interfere with smokers’ perceptions of the messages being mixed or conflicting. Future research should vary the content and number of messages to better understand the message effects. Notably, our findings about the interaction between pretest efficacy beliefs, message condition, and need for closure had a small effect size (*r* = 0.06). The effect size is comparable with other communication research. For example, about 25% of communication studies have effect sizes smaller than *r* = 0.10 [68]. Media campaigns for smoking prevention and cessation have an average effect size of *r* = 0.03 [69]. Given tobacco messages could be seen by a large number of people, small message effects still deserve merit [70]. However, the small effect size of our findings might also suggest the presence of some methodological limitations we identified above, which highlights the importance of conducting replication research beyond our initial study.

Besides encouraging future replication studies, our study points out several future research directions. First, given nonsmokers are also an important population in evaluating MRMs, future research should examine our research questions among nonsmokers. Additionally, future research may want to manipulate need for closure through situational cues (e.g., time for information processing or mental fatigue) to better explore the role of need for closure in response to MRMs with a warning or other conflicting e-cigarette messages. Additionally, future research should examine the impact of MRMs with negative health warnings in other formats (e.g., marketing brochure, package labeling, fact sheet), with other designs, and for other potentially less harmful tobacco products. In sum, studying the effects of communication about potentially less harmful tobacco products is important but challenging. Because the current communication environment about e-cigarettes contains mixed information [9,69,71,72], it is critical to build upon the line of research on the effects of mixed e-cigarette messages using a motivational cognition framework.

## 5. Conclusions

This study tested how exposure to e-cigarette modified risk messages (MRMs) with a nicotine addictiveness warning would impact smokers’ posttest efficacy regarding switching completely to e-cigarettes by applying a motivational cognition framework involving biased assimilation, attitude polarization, and need for closure. Our findings did not support the notion that MRMs with a nicotine warning would polarize smokers who initially held differing efficacy beliefs about the efficacy of switching completely to e-cigarettes in reducing smoking-related risks. Nonetheless, we found that need for closure and people’s initial efficacy beliefs worked in tandem to influence message response: MRMs with a nicotine warning only enhanced efficacy beliefs of smokers with low initial efficacy beliefs and low need for closure. Consistent with a recent study [25], this suggests the importance of considering individuals’ motivational and cognitive differences in the assessment of mixed communication surrounding e-cigarettes.

## Figures and Tables

**Table 1:** Characteristics of study participants.

	Overall (*n* = 761)
**Gender**	
Male	48.0
Female	51.5
Transgender	0.5
**Age**	
18–29	22.5
30–44	33.0
45–59	25.1
60+	19.4
**Race**	
White	73.9
Black or African American	15.2
American Indian or Alaska Native	1.4
Asian	3.8
Other	5.7
**Ethnicity**	
Hispanic	12.4
Non-Hispanic	87.6
**Education**	
Less than high school	8.1
High school	31.5
Some college	33.0
Bachelor or higher degree	27.3
**Daily Smoker**	
Yes	63.3
No	36.7
**E-Cigarette Use**	
Current	46.4
Former	24.4
Never	29.2
**Current Cigarette Smoking**	
Yes	95.9
No, former smoker	4.1
**Tried to Quit in the Past 12 Months**	
Yes	52.6
No	47.4

**Table 2:** Regression analyses predicting posttest perceived efficacy of switching completely to e-cigarettes.

Predictors	Unstandardized Coefficient	Standard Error	Part Correlation	*p* Value
Male (vs. other)	−0.07	0.12	−0.01	0.56
Age	**−0.01**	**0.00**	**−0.06**	**0.01**
Non-Hispanic White (vs. Non-Hispanic other)	−0.25	0.23	−0.03	0.28
Non-Hispanic Black (vs. Non-Hispanic other)	−0.10	0.27	−0.01	0.70
Hispanic (vs. Non-Hispanic other)	−0.29	0.27	−0.03	0.29
Below college education (vs. other)	−0.05	0.12	−0.01	0.68
Current e-cigarette users (vs. never)	0.15	0.16	0.02	0.35
Ever but not current e-cigarette users (vs. never)	0.09	0.16	0.01	0.57
Nicotine dependence	**0.10**	**0.04**	**0.05**	**0.02**
Past quit attempt (vs. no)	0.19	0.13	0.03	0.14
Ever switch to a lower tar or nicotine cigarette (vs. no)	**0.34**	**0.13**	**0.06**	**0.01**
Modified risk messages with a nicotine warning (MRMs vs. control)	**0.12**	**0.04**	**0.07**	**0.00**
Pretest perceived efficacy (Efficacy)	**0.78**	**0.05**	**0.38**	**0.00**
Need for closure (NC)	0.07	0.12	0.01	0.57
Efficacy × MRMs	**−0.04**	**0.02**	**−0.06**	**0.01**
Efficacy × NC	−0.06	0.05	−0.03	0.19
MRMs × NC	−0.01	0.04	−0.00	0.86
Efficacy × MRMs × NC	**0.04**	**0.02**	**0.06**	**0.01**
Total *R^2^*	**0.60**			
Adjusted *R^2^*	**0.59**			

Note. Bold numbers represent significance at *p* < 0.05.

## Data Availability

The data are available upon reasonable request.

## Supplementary Materials

The following are available online at https://www.mdpi.com/article/10.3390/ijerph18116094/s1, Figure S1: Message stimuli.
