# Supplementary material for: Will E-Cigarette Modified Risk Messages with a Nicotine Warning Polarize Smokers’ Beliefs about the Efficacy of Switching Completely to E-Cigarettes in Reducing Smoking-Related Risks?
Source: Int J Environ Res Public Health. 2021 Jun 5;18(11):6094. doi: 10.3390/ijerph18116094 (PMC8200968; doi:10.3390/ijerph18116094)
Supplement: Supplementary file 1 [file ijerph-18-06094-s001.zip › ijerph-1185484-supplementary.pdf]

Supplementary Material

Figure S1. Message Stimuli.

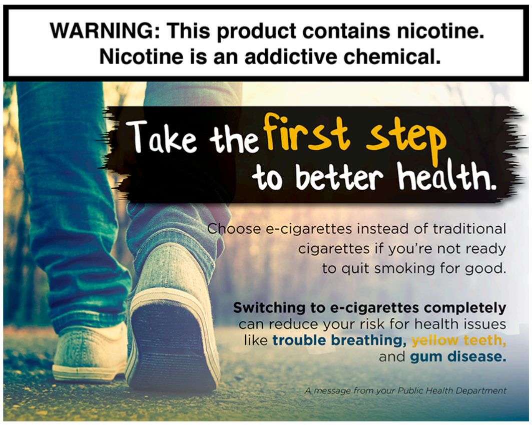

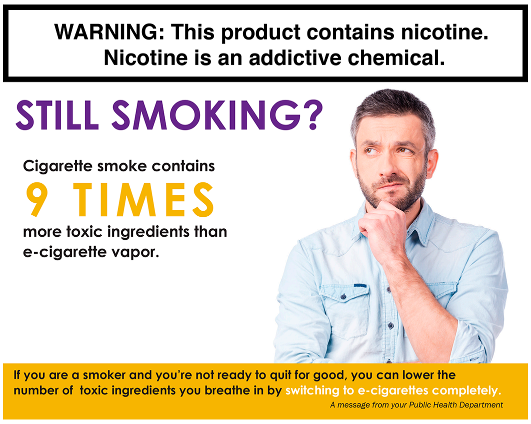

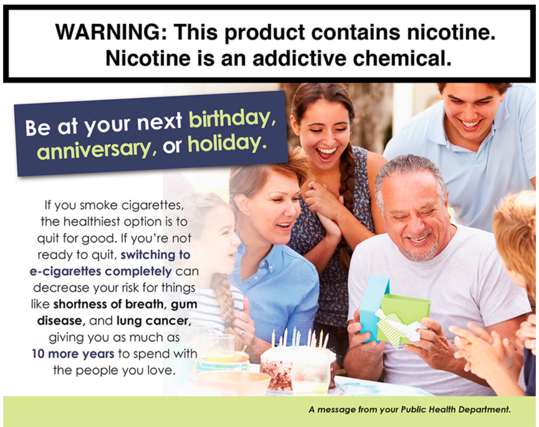

(modified risk messages with a warning)

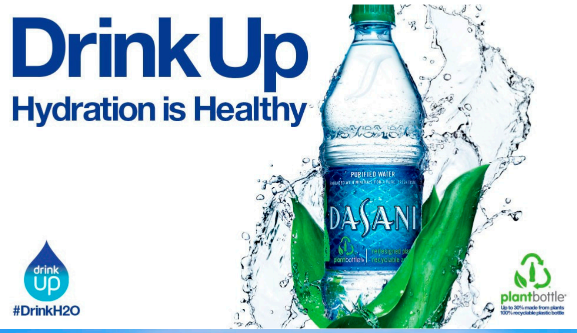

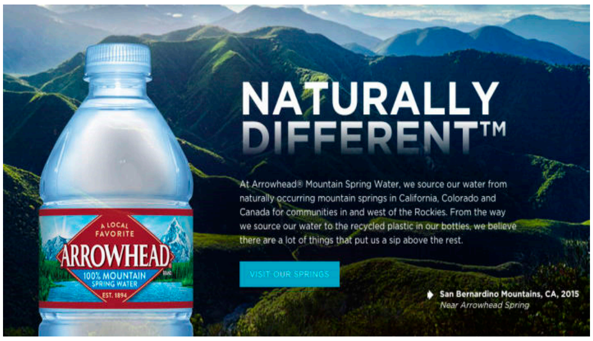

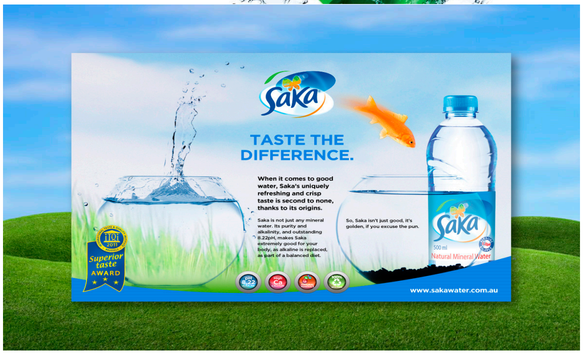

(three control messages)
